# Significant Anticancer Activity of a Venom Fraction Derived from the Persian Gulf Sea Anemone, *Stichodactyla haddoni*

**DOI:** 10.22037/ijpr.2019.14600.12521

**Published:** 2020

**Authors:** Ziba Moghadasi, Delavar Shahbazzadeh, Shahla Jamili, Nariman Mosaffa, Kamran Pooshang Bagheri

**Affiliations:** a *Venom and Biotherapeutics Molecules Lab., Medical Biotechnology Dept., Biotechnology Research Center, Pasteur Institute of Iran. Tehran, Iran. *; b *Iranian Fisheries Science Research Institute, Agricultural Research, Education and Extension Organizatio,Tehran,Iran. *; c *Department of Immunology, Faculty of Medicine, Shahid Beheshti University of Medical Sciences. Tehran, Iran.*

**Keywords:** Anticancer protein, Venom, The Persian Gulf, Sea anemone, *Stichodactyla haddoni*

## Abstract

Chemotherapy is still one of the main therapeutic regimens in cancer patients but its toxicity is a hard challenge for every patient yet. One of the available solutions is tracing for non-toxic anticancer agents from natural resources. Numerous proteins and peptides in the venom of sea anemones are potentially useful agents with pharmacological properties. Concerning to significance of this issue, the current study was aimed to finding a non-toxic anticancer fraction from the venom of the Persian Gulf sea anemone, *Stichodactyla haddoni*. Anticancer and hemolytic activity of crude venom was evaluated and followed by fractionation using RP-HPLC. Breast, Brain, and Colon cancer cell lines were selected to assessment of anticancer activity and toxicity. IC50 of crude venom on the abovementioned cancer cell lines was as 4.13, 6.58, and 31.54 µg, respectively. According to the results obtained by paired sample t-test and comparison of toxicity of the fractions in normal cell line, F10, designated as hadonin, was determined as the candidate anti-cancer fraction. The non-toxic dose of F10 was 20 ng in which showed respectively 66, 29, and 7 anticancer activities on breast, brain, and colon cancer cell lines. According to results, anticancer activity of hadonin is of high pharmaceutical value to follow its therapeutic potency in animal model. In conclusion, the venom of the Persian Gulf sea anemone contains a potential anticancer agent with reasonable activity at nanogram level against three kinds of cancer cells with no toxicity on normal cells.

## Introduction

Cancer is globally still a main public health concern. According to the data collected by the International Agency for Research on Cancer (IARC) and World Health Organization, 8.2 million People (13%) die each year from cancer worldwide.

Breast, colon, and brain cancers are of high significance regarding the clinical signs, prevalence, complications of anticancer drugs, and treatment failures (1, 2). Although chemotherapy is still one of the main solutions but following major clinical complications is being happed consequently due to their toxicity (3).

Regarding this vital condition, discovery or design of a non-toxic anticancer drug is being necessitated. One of the available solutions is tracing for non-toxic agents from natural resources.

Numerous proteins, peptides, and chemical agents in the venom of venomous marine animals are potentially useful biologically active molecules with pharmacological properties. During the past decade, many studies have been focused on tracing of novel drugs from marine animals (4-15). 

Among many marine derived potential drugs, some of them including a series of anticancer drugs like brentuximab vedotin (Adcetris^®^) (16), trabectidin (Yondelis®) (17), cytarabine (Cytosar-U^®^, Depocyt®) (18, 19), and vidarabine (Vira-A®) (11, 20) are approved and currently used in the market. These agents are significant successful examples of marine drugs.

A series of drug candidates derived from marine invertebrate and vertebrate animals are in different phases of clinical studies including Plitidepsin, bryostatin, hemiasterlin, and elisidepsin against lymphoma, leukemia, colon, breast, and various cancers (21-24).

Recently, Ramezanpour et al, in 2012 reported anticancer effects of the venom of some sea anemones *H. crispa*, *H. magnifica*, *E. quadricolor*, *C. adhaesivum*, and *H. malu* against three human cancer cell lines (25). Monroy-Estrada et al demonstrated synergistic effect of sea anemone *Bunodeopsis globulifera* isolated fractions on human lung adenocarcinoma cells (26). Soletti *et al*., in 2008 showed that the cytolysins derived from sea anemones potentiated the cytotoxicity induced by low-dose concentrations of the chemotherapeutic drugs, 10-300-fold less of the therapeutic drug (27). Sea anemones venoms contain different cytolytic agents (28). Cytolysis may induce cell damage in different normal and cancer cells that would be related to difference in cell membrane characteristics (29, 30). Many studies have been demonstrated that cytolytic peptides can induce more damage in cancer cells than normal cells (31, 32). These peptides have been proposed for cancer therapy against human adenocarcinoma cells like breast, lung, stomach, and other cancers (26, 33, 34).

There is no report regarding an anticancer non-toxic agent from marine venomous or common creatures. To reach this goal, many natural molecules should be purified and examine on a variety of cancer and normal cell line as a screening approach. 

Among sea anemones, *Stichodactyla haddoni* from the Persian Gulf was selected for tracing anticancer non-toxic agents. *S. haddoni* is an ocean dwelling sedentary organism belonging to the family of Stichodactylidae. The venom of this family could be a potential source of bioactive pharmaceutical compounds (14, 35-37). 

Variation in geographical distribution of similar venomous animals could lead to variation in biological activities and toxicity. Particular condition of the Persian Gulf as a closed important ecosystem is a good opportunity to study biological activities and toxicity in venomous animals.

Regarding the significance of searching for a non-toxic anticancer agent, the goal of the current study was aimed to determine the anticancer activities of all HPLC purified fractions from the Persian Gulf sea anemone, *Stichodactyla haddoni* on different cancer cell lines including Breast cancer (MD-MBA231), Glioblastoma (U87mg), Colon cancer (SW948), as well as study of their toxicity. 

## Experimental

*Reagents and media*

3-(4,5-dimethylthiazol-2-y1)-2,diphenyltetrazolium bromide (MTT) [Sigma], bicinchoninic acid solution (BCA) [iNtRON Biotechnology Co. South Korea], Triton purchased [Sigma–Aldrich Co], Dimethyl Sulfoxide (DMSO) [Merck Company, Germany]. RPMI-1640 medium [Gibco Company, USA], DMEM medium [Gibco Company, USA], Fetal bovine serum (FBS) [Gibco Company, USA], Penicillin /Streptomycin [Gibco Company, USA], Trypsin–EDTA [Gibco Company, USA], Trypan blue [Sigma], Methanol [Merck ,Germany].

*Sample collection*

One sample of *Stichodactyla haddoni* weighted 85.82 gram was collected manually at depth of 30m from coastal waters of Lark island, Persian Gulf (the south of Iran) in August 2015. The specimens were kept in -20 °C and transferred to Venom and Biotherapeutics Molecules Laboratory (VBML) at Pasteur Institute of Iran, Tehran. 

*The algorithm of study design*

Our final objective was reaching a non-hemolytic non-toxic anticancer fraction against examined cancer cell lines. The following chart is described as classified goals in each step (Figure 2). 

*Venom extraction and preparation*

The frozen specimens thawed in room temperature and surface mucus layer was cleaned. Tentacles were removed from base by a sterile scalpel, trimmed to small pieces, and divided to two equal parts weighted 8.96 grams for venom extraction. Macroscopic and microscopic view of tentacles is showed in Figure 3.

*Water extraction*

Sixty nine mL sterile deionized water added to 8.96 grams of fragmented tentacles, mixed in a vortex and incubated at 4 °C for 24 h. The solution was centrifuged (Sigma 1-14, Germany) at 10625 g for 10 min. Supernatant lyophilized in a freeze dryer system (Alpha 1-2 LD plus, Martin Christ Gefriertrocknungsanlagen Co., Germany). The powder resuspended in sterile water for injection and stock solution maintained at -70 °C. 

*Protein estimation and SDS-PAGE*

Concentration of crude venom was determined by bicinchoninic acid (BCA) protein assay method according to manufacturer instructions (iNtRON Biotechnology Co. South Korea). Sodium Dodecyl Polyacrylamide Gel Electrophoresis (SDS-PAGE) was carried out according to standard method (38). The fractions were loaded onto a 12% polyacrylamide gel and stained with Coomassie brilliant blue. 

*Hemolysis assay for water extract of crude venom*

This assay was performed as described before (39). Fresh human blood was drawn by venous puncture using heparinized tubes. Erythrocytes were washed three times with phosphate-buffered saline (pH = 7.4) and a suspension of 2% RBC was prepared in normal saline. 

Serial amounts of venom prepared in a microplate ranged from 100 to 0.78 µg in 100 µL normal saline and 2% RBC suspension (100 µL) added to each well and incubated for 1 h at 37 °C. The samples were than centrifuged for 10 min at 1664 g (Sigma 3-18k), and the absorbance of the supernatant was determined at 540nm in a microplate spectrophotometer (EPOCH, BioTeK Co., USA). Normal saline and triton X100 were used as negative and positive control respectively. The percent of hemolysis was calculated as follows: 

$\mathrm{Hemolysis}\left(\%\right)=\left[\frac{\mathrm{OD}\mathrm{test}-\mathrm{OD}\mathrm{negative}\mathrm{control}}{\mathrm{OD}\mathrm{positive}\mathrm{control}-\mathrm{OD}\mathrm{negative}\mathrm{control}}\right]\times 100$

 Equ.1

*Anticancer activity of crude extract against cancer and normal cell lines*

*Cell Culture*

The breast cancer cell line (MDA- MB-231), colon cancer cell line (SW948), brain cancer cell line (U87mg) and Normal Human Dermal Fibroblast (NHDF) line were purchased from Pasture Institute of Iran. MDA- MB-231 and SW948 cell lines were cultured in RPMI 1640 (Gibco Co., USA) whereas U87mg and Human fibroblast cell lines in DMEM (Gibco Co., USA). The cells were seeded in tissue culture flasks with 10% FBS and 100U/microgram penicillin/streptomycin and incubated at 37 °C, 5% CO2, and 80% humidity. 

Breast Cancer, Brain Cancer, Colon Cancer, and Normal Human Fibroblast were abbreviated as “BC”, “BrC”, “CC”, and “n” respectively. 

*Cytotoxicity assay*

The cytotoxicity effects of crude extract were determined using MTT assay (40). The cells were seeded at 1 × 10^4 ^cells/well in culture medium (100µL) supplemented with 10% FBS. After 24 h incubation (37 °C, 5% CO2), cells were washed with PBS. Then the fresh media with 10% FBS (100 µL) was added to each well. The cells exposed to serial amounts of crude venom ranged from 100 to 0.78 µg and incubated for 24 h at 37 °C. The cells with culture medium alone were considered as negative control. 

MTT (5 mg/mL in PBS) was added to each well (10 μL MTT/90 μL medium) and the plates were incubated at 37 °C for 4 h and then 100 μL DMSO was added to each well. The plates incubated at room temperature in dark room for 10 min with shaking and finally the absorbance was measured on a microplate spectrophotometer (EPOCH, BioTeK Co., USA) at 570 nm. The percentage of cell death was calculated according to the below formula: 

$\mathrm{Dead\%}=\left[1-\left(\frac{\mathrm{OD test}}{\mathrm{OD control}}\right)\times 100\right]$

Equ. 2

*Fractionation of crude venom by RP-HPLC*

The lyophilized venom was dissolved in distill water (100 µL) and insoluble material discarded by centrifugation at 10625 g for 10 min. The protein fractions were separated using a HPLC instrument (Knauer-Germany). Fifty µL of the prepared venom (8 µg/µL) manually injected to C18 column (5µm, 100A, 250 4.6mm) and eluted in a linear gradient of acetonitrile containing 0.5% TFA (solution B) and 0.5% TFA in water (solution D) at flow rate 1 mL/min. The resultant peaks were detected at 214nm and 280nm, and were collected manually. The obtained fractions were lyophilized in a freeze dryer system (Alpha 1-2 LD plus, Martin Christ Gefriertrocknungsanlagen Co., Germany). The powder resuspended in sterile water for injection and stock solution maintained at -70 °C. Concentration of the fractions was estimated as detailed above. 

*Hemolysis assay for HPLC collected fractions*

Hemolytic activity was performed as detailed above with some modification. Briefly, erythrocytes were prepared as above and a suspension of 0.5% RBC was prepared in normal saline. Serial amounts of fractions prepared in a micro plate ranged from 1.56 to 0.01 µg in 100 µL normal saline and 0.5% RBC suspension (100 µL) added to each well and incubated for 1 h at 37 °C. The other steps were done as outlined before and the percent of hemolysis was calculated according to above formula. 

*Anticancer evaluation of hemolytic and non-hemolytic fractions*

The breast cancer cell line (MDA- MB-231), colon cancer cell line (SW948), brain cancer cell line (U87mg), and NHDF cell line were cultured and incubated as described before. The cytotoxicity effects of HPLC fractions were determined using MTT assay (40). All conditions were similar to MTT assay for crude venom with the exception of amounts that ranged from 1.56 to 0.01 µg. 

*Statistical Analysis*

Variance of hemolytic activity for examined doses was determined using one sample *t*-test. For estimation of correlation between hemolytic activity of crude venom and doses, linear regression test was performed.

To control the variance of examined doses in anticancer assay of the crude, one sample *t*-test was carried out. Paired sample student *t*-test was performed to cross comparison of anticancer activities of crude venom between BC and BrC, BC and CC, BrC and CC cell lines. Linear regression test was done to evaluate the correlation between anticancer activity and examined doses between cancer and normal cells as well as Regression between cancer cells.

Variance of examined doses in hemolysis assay of the collected fractions was checked by one sample *t*-test. Linear regression test was done to evaluate correlation between hemolysis activity of the fractions and examined doses. 

 Difference of anticancer activity between the fractions, against each cancer cell line, was estimated by paired sample *t*-test. Furthermore, Comparison of toxicity of non-hemolytic fractions on normal cell line was evaluated by paired sample *t*-test. 

Significance between anti-cancer activity of the candidate fraction on Brain, breast, and colon cancer cell lines and NHDF cell line was compared by paired sample student *t*-test. To evaluate the similarity of anticancer activity of candidate fraction on breast, brain and colon cancer cell lines, linear regression test was done. In order to determine the candidate anticancer dose, toxicity of examined doses on cancerous and normal cell lines was compared point to point together. 

*Assessment of the purity for purified candidate fraction by SDS-PAGE*

To control the purity of eluted candidate fraction, the resultant peak was collected and freeze dried in a freeze dryer system (Alpha 1-2 LD plus, Martin Christ Gefriertrocknungsanlagen Co., Germany). The powder resuspended in HPLC grade water and its concentration was determined by BCA assay. The fraction was loaded onto a 12% polyacrylamide gel and stained with Coomassie brilliant blue as noted above.

## Results

*Protein profile of crude venom*

SDS-PAGE results showed about 12 separate bands and molecular weight of observed proteins ranged approximately from 8 to 250 kDa (Figure 4). 

*Hemolysis assay for water extract of crude venom and statistical analysis*

The amount of 25 µg crude venom produced 100% hemolysis and HD50 identified at 0.5 µg. Based on regression analysis, no general linearity was seen at examined amounts of venom ranged from 0.023 to 25 µg (R^2^= 0.299) (Figure 5). 

Analysis of variance for hemolysis activity showed that in almost all doses, the activities were similar (*P* value < 0.001). According to linear regression assay no significant correlation was observed between hemolytic activity and doses (R^2 ^= 0.3).

As showed in Figure 5 correlation of activity and doses were visually distinguishable at two distinct ranges so further regression analyses were performed to better understanding of trends. 

Slope of hemolysis activity intensely increased from 3.13 to 77.2% that this raising trend corresponded to amounts ranged from 0.023 µg (23 ng) up to 0.39µg (390 ng) (R^2^= 0.978). Slope of hemolysis activity gradually raised from 83.6 to 100% that was corresponded to the amounts ranged from 0.78 to 25 µg. 

*Anticancer activities of the crude extract against cancer and normal cell lines and statistical analysis*

IC50 of crude venom against BC, BrC, CC, and normal cell lines was observed at 4.13, 6.58, 31.54, and 93.45 µg respectively. Analysis of variance for anticancer activity of crude venom on BC, BrC, and CC cell lines showed that activity was similar together in almost all doses (*P* value < 0.001). Cross comparison of anticancer activities of crude venom between BC and BrC, BC and CC, BrC and CC cell lines showed significant difference of anticancer activity by Paired sample student *t*-test respectively (*P* value 0.001, < 0.001, and 0.002). 

Comparing the induced toxicity of crude venom between BC, BrC, and CC cell lines and NHDF cell line (n) showed a significant difference (*P* value < 0.001) and also toxicity on all three cancer cell lines were very greater than normal cells. Based on linear regression analysis, cross comparison of anticancer activity of crude venom between BC and BrC, BC and CC, BrC and CC cell lines showed significant correlation respectively (R^2^=0.984, 0.945, 0.944). 

Linear regression analysis showed significant correlation between anticancer activity of crude venom on BC, BrC, and CC with normal cell lines by increasing examined doses (R^2^_BC _= 0.692, R^2^_BrC_ = 0.754, R^2^_CC_ = 0.791, R^2^_n_ = 0.811). 

*Fractionation of crude venom by RP-HPLC*

HPLC chromatography of *S. haddoni* venom on C18 column resulted in 11 fractions. The obtained fractions were quantified by the BCA method. Among eluted peaks, 11 peaks with area percent of at least 3% were selected for the next experiments. The aliquots of each fraction were used for examining hemolytic and anticancer activities. Area percent of each fraction is showed in Figure 7 Almost all fractions were eluted up to about 23 min. The major fractions were hydrophilic or very weak hydrophobic characteristics. 

*Hemolysis assay for collected fractions and statistical analysis*

The highest activities of the first six fractions on human RBCs were equal to 30, 31, 28, 8, 4, and 6% at 1.56 µg, respectively. The fractions from F7 to F11 showed no hemolytic activity at all amounts (Figure 8). Regression analysis showed significant correlation between hemolysis activity of each fraction and examined doses (R^2^_F1 _= 0.84, R^2^_F2_ = 0.97, R^2^_F3_ = 0.71, R^2^_F4_ = 0.83, R^2^_F5_ = 0.79, R^2^_F6_ = 0.96). 

The highest activities of the first six fractions on human RBCs were equal to 30, 31, 28, 8, 4, and 6% at 1.56 µg respectively. The fractions from F7 to F11 showed no hemolytic activity at all amounts. Regression analysis was showed significant correlation between hemolysis activity of each fraction and examined doses (R2F1 = 0.84, R2F2 = 0.97, R2F3 = 0.71, R2F4 = 0.83, R2F5 = 0.79, R2F6 = 0.96).

*Correlation of hemolytic activity and hydrophobicity*

Based on the order of elution time in which the fraction eluted from the column, hemolysis activity was descended significantly (Figure 9A). 

According to regression analysis, hemolysis activity of the eluted fractions were inversely correlated with increasing the acetonitrile percent (R^2^ = 0.789). The subsequent fractions (F7-F11) eluted by acetonitrile ranged from 9.22% to 15.07 and had no hemolytic activity (Figure 9B). 

*Anticancer activity of isolated fractions on three cancer cell lines*

F1 had the greatest anticancer activity against breast cancer (98%) and was recorded at 1.6 µg. The highest anticancer activity induced by F4 on both brain and colon cancer cells were showed in 87 and 69%, respectively at 1.6 µg. The lowest activity against breast, brain, and colon cancer cells were induced by F9, F5, and F10, respectively (Figures 10, 11 and 12). 

The slope of anticancer activity was intensely raised up to 0.4 µg for almost all fractions against all three cancer cell lines and trend of activity was dose dependent. This trend was gradually tended to rising or to be constant while the doses are being increased (Figure 10, 11 and 12). 

Anticancer activity of examined doses of all fractions against breast, brain, and colon cancer cells were correlated significantly together by linear regression analysis (R^2^_BC_= 0.529 - 0.95, R^2^_BrC_= 0.679 - 0.868, R^2^_CC_= 0.528 - 0.945). 

*Determination of candidate fraction by statistical analysis*

Analysis of variance for toxicity of non-hemolytic fractions on normal cells (F7, F8, F9, F10, F11) showed that activity of each dose of each fraction was totally the same as the other one (*P* value for F7-F11< 0.05). It means that toxicity of higher and lower doses are totally the same but it is still a chance to finding a non-toxic dose. 

Paired sample student *t*-test showed significant difference between toxicity of F9, F10, and F11 fractions on normal cell line (*P* value < 0.05) while F7 and F8 had similar toxicity with F10 and F11, and F9, respectively (*P* value > 0.05). 

Comparison of toxicity of F7, F8, F9, F10, and F11 on normal cell with the other cancer cells showed a significant difference based on paired sample student *t*-test (*P* value < 0.05). Comparing the mean toxicity between these fractions showed that F10 had the least toxicity (Mean: 11.75% ± 3.7) (Figure 13). 

Linear regression analysis showed significant correlation between toxicity of F7, F8, F9, F10, and F11 on normal cell lines with increasing doses of the fraction (R^2^_F7 _= 0.691, R^2^_F8 _= 0.736, R^2^_F9 _= 0.772, R^2^_F10_ = 0.759, R^2^_F11_ = 0.736) (Figure 14). 

F10 induced significant different toxicities when used against three cancer cell lines (*p* value <0.001) and also comparison of toxicity of F10 between cancer and normal cell lines showed significant difference (*p* value <0.001) according to paired sample *t*-test analysis. 

According to linear regression analyses, toxicity of F10 on all three cancer cell lines (R^2^_BC _= 0.725, R^2^_BrC _= 0.744, R^2^_CC_ = 0.847) compared to normal cell line (R^2^_n _= 0.759) was completely dose dependent (Figure 15).

*Determination of the candidate dose*

The candidate dose was determined at 20 ng by point to point comparison of toxic activity of F10 at the examined doses on breast, brain, and colon cancer cell lines reference to normal fibroblast cell line. According to results, F10 at 20 ng showed respectively 66, 29, and 7 anticancer activities on breast, brain, and colon cancer cell lines. At this amount no toxicity was observed on normal fibroblast human cell line (Figure 16).

*Assessment of the purity for purified candidate protein by SDS-PAGE*

Candidate protein was subjected for electrophoresis for controlling the purity. SDS-PAGE showed one pure band corresponding to molecular weight approximately at 17.5 kDa (Figure 17).

## Discussion

Natural products play an apposite role in cancer therapy today with existing numbers of anticancer agents used in the clinic being either natural or derived from natural products from various sources particularly marine organisms. Tracing for novel drugs is still important for cancer therapy, caused the fast development of anticancer drugs. 

High toxicity of almost all of cancer chemotherapy drugs has triggered the request for novel anticancer drugs with little side effects and or with greater therapeutic efficiency. 

Hemolysis activity of the crude was generally not dose dependent at estimated range (R^2^= 0.299) (Figure 5). According to calculated R^2^ high linearity that was seen at the range of 0.023 µg (23 ng) up to 0.39 µg (390 ng) (R^2^= 0.978) indicated strange linear trend of activity. Although there is a lack of correlation between hemolytic activity and doses (R^2 ^= 0.3), a relative meaningful correlation about 81% is considerable to describe dose dependent trend of activity (P value 0.08). In reference to calculated R^2^, high linearity was seen at examined range of doses (R^2^= 0.914). This result demonstrates that hemolytic activity strangely was linear at the range of 0.78 to 25 µg.

Hemolysis activity on human erythrocytes was not seen in dose dependent manner at estimated doses (R^2^=0.299) but at two distinct ranges of 0.023-0.39 µg (R^2^=0.978) and 0.78-25 µg (R^2^=0.914) activity was dose dependent and linearity was significant. Similarly, Subraminan *et al*. in 2011 showed the hemolytic activity of *P. indicus* and *P. sinensis* against chicken erythrocytes and also *H. magnifica* and *S. hadonii* against chicken and goat erythrocytes (41). Sudharsan *et al.* in 2013 reported hemolytic activity of *S. mertensii* methanolic crude venom on chicken and human erythrocytes too (42). 

Marino *et al.* in 2009 showed that the crude venom from the sea anemone *Aiptasia mutabilis* had dose-response hemolytic activity against human erythrocytes (43) but our results disagree with that. 

Amazing Similar activity of crude venom on BC, BrC, and CC cell lines based on variance analysis (*p* value <0.001), not only may give us a chance to finding an anticancer fraction but may boost our chance to apply lower doses of anticancer agent and avoiding toxicity in normal cell line. The results obtained from Cross comparison of anticancer activities of crude venom between BC and BrC, BC and CC, BrC and CC cell lines showed that each examined cancer cell line has different sensitivity when exposed to the same crude venom. Crude venom induced more lethality on BC cells than the other cancer cell lines. Significant difference in toxicity of crude venom against examined cancer cell line compared to normal cell line (*p* value <0.001) is an interesting phenomenon pointed out to existence of a non-toxic anticancer fraction in crude venom. According to significant cross-correlations between anticancer activity of crude venom on BC and BrC, BC and CC, BrC and on CC cell lines, similar mechanism of toxicity would be contemplated. In feference to significant correlation between anticancer activity of crude venom on BC, BrC, and CC with NHDF cell lines by increasing examined doses, this similarity would suggest a common dose dependent trend of toxicity on all three cancer cell lines and normal cells as well.

Cline *et al.* in 1995 pointed out cytotoxic effect of crude extract from the sea anemone *Urticina piscivora and showed that *the growth of 50% of oral human epidermoid carcinoma cells (KB), mouse lymphocyte leukemia cells (L1210), and human embryonic lung diploid cells (HEL299) were inhibited at 6.54, 10.07, and 2.34 mg/mL, respectively (44). Our study also demonstrated anticancer activity of *S. haddoni* crude extract. 

The crude venom extracted from* H. malu*, *C. adhaesivum* and *E. quadricolor* had a significant inhibitory effect on skin cancer, A431 cells. Furthermore, *H. malu* and *C. adhaesivum* had a significant inhibitory effect on breast cancer, T47D cell line at 40 μg/mL (25). This result was in accordance with toxicity of *S. haddoni crude* venom on breast cancer cell line, MDA-MB231 approximately.

According to observed retention time, almost all of the fractions were relatively hydrophobe and eluted from 1.357 to 17.8% acetonitrile. The first three fractions were eluted at 2.758, 3.732, and 4.739 min referring to hydrophilic proteins or peptides. The largest peak was seen at 6.357 min representing a very weak hydrophobic fraction eluted at 1.357% acetonitrile.

According to significant correlation between hemolysis activity of all hemolytic fractions and examined doses, activity was indicated as dose dependent and a linear trend of activity was showed. Similar trend of hemolysis would be indicated to the same mechanism induced by the same hemolytic agent. 

According to analysis of variance for all hemolytic fractions, similar activity at all doses of each fraction was showed (*p* value < 0.05). Comparing the mean activity of all fractions together by paired sample *t*-test showed significant difference between them (*p* value <0.05) except for the following pairs including F1-F2, F1-F3, F2-F4, F2-F5, and F5-F6 (*p* value >0.05). 

Analysis of correlation between hemolysis activity and examined doses by linear regression test indicated that all fractions had dose dependent manner. Cross comparison of trend of hemolysis in all hemolytic fractions was performed by linear regression test and the results showed similar trend of activity in all fractions (R^2 ^= 0.74-0.999). 

Ravindran in 2010 showed hemolytic activity in all fractions isolated from three sea anemone including *Heteractis magnifica*, *S. haddoni*, and *Paracodylactis sinensis* collected from Indian costal region. Interestingly, some of our HPLC isolated fraction had no hemolytic activity (45). 

During data interpretation regarding hemolytic activity of the isolated fractions, a hypothesis was triggered concerning the correlation of hemolytic activity and hydrophobicity. Following further analysis by linear regression test, a significant correlation was showed between activity of hemolytic fraction and the percent of acetonitrile in which the fractions were eluted. 

This issue indicated that hydrophobic fractions have lower toxicity against human RBCs. Interestingly the first three hydrophilic fractions had greater toxicity than the subsequent weak hydrophobic fractions demonstrating that hydrophobicity of S. haddoni fractions is in accordance with no hemolytic activity. 

Analysis of variance for anticancer activity of isolated fractions on cancer cells showed that activity of each dose of each fraction was generally similar together (*p* value of all fractions: < 0.01).

Cross comparison of the mean of anticancer activity of each fraction with the other fractions by Paired sample *t*-test showed significant differences in almost all isolated fractions against BC, BrC, and CC (*p* value < 0.05) in exception of some fractions. In reference to the difference of hydrophobicity in isolated fractions (Figure 6) and difference in their anticancer activity, it could be suggested that each isolated fraction has a distinct entity or mechanism.

Anticancer activity of examined doses of all fractions against breast, brain, and colon cancer cells were correlated significantly together by linear regression analysis (R^2^= 0.528 - 0.95).

Significant difference between toxicity of F9, F10, F11 fractions on normal cell line pointed out to distinct entity and to different mechanism of toxicity. 

According to significant difference between toxicity of F9, F10, and F11 on normal and cancer cells, F10 was selected as our candidate fraction since it had the lowest toxicity on normal cells.

Comparison of trend of toxicity for F9, F10, and F11 fractions showed a similar behavior and this issue suggested that these three fractions have possible similar mechanism of toxicity. 

Since the mean toxicity of F10 on the cancerous cells is remarkably upper than the normal cells, dose dependency of F10 provided us with a good chance to access a non-toxic dose on normal cell. Furthermore, it means that lower dose of F10 at nanomolar scale avoiding immune system to react against the molecule as immunogen.

Cline *et al.* in 1995 demonstrated anticancer effect of a protein designated UpI isolated from the sea anemone *Urticina piscivora *and showed that the growth of 50% of oral human epidermoid carcinoma cells (KB), mouse lymphocyte leukemia cells (L1210), human embryonic lung diploid cells (HEL299) were inhibited at 40.32, 29.99, and 29.74 mg/mL respectively (44). UpI was found to be hemolytic on human RBC but hadonin showed no hemolytic activity in our study.

In reference to Jiang *et al.* study in 2003, a recombinant protein, Src I, from the sea anemone *Sagartia rosea* exhibited 50% cytotoxic activity on human cell lines, including astrocytoma (U251) (3.5 μg/mL), Non-Small Cell Lung Cancer Carcinoma (NSCLC) (2.8 μg/mL), liver carcinoma (BEL-7402) (3.6μg/mL), stomach adenocarcinoma (BGC-823) (7.4 μg/mL), NIH Swiss mouse embryo (NIH/3T3g) (3.4 μg/mL) (34). Src I as an anticancer agent had been showed hemolytic activity and in fact, was found to be strongly toxic (HD50 at 0.43 μg/mL) but hadonin showed no hemolytic activity in our study. 

Avila *et al.* in 1988 isolated a toxin from the sea anemone *Stoichactis helianthus *that exhibited toxicity on HL-60 human myelocytic leukemia cells (46) but also was toxic against human erythrocytes and human peripheral mononuclear cells too while hadonin induced no toxicity on human RBCs and human normal fibroblast cells as well. 

Fedorov *et al.* in 2010 isolated an anticancer protein, RTX-A, from the sea anemone *Heteractis crispa. *RTX-A reduced cell viability of JB6 P+ Cl41 cells, Hela, THP-1, MDA-MB-231 and snu-c4 human tumor cell lines (33). 

EqTX-I cytolysis toxin from the sea anemone *Actinia equina* also induces a decrease in the viability of V-79-379 A cells (diploid lung fibroblast from Chinese Hamster) in a concentration-dependent manner (47) while hadonin showed exceptional non-toxic activity on human normal fibroblast cell line. 

EqTx-II from *Actinia equina* was studied for cytotoxicity against human glioblastoma U87 and A172 cell lines. After 24 h of treatment, 10 mg/mL EqTx-II showed remarkably cytotoxic and reduced the viability of U87 and A172 cells to 60 and 48%, respectively, but was toxic on normal cells at 10 mg/mL and decreased the viability to 80% (27) while hadonin showed anticancer activity on the examined cancer cell lines but non-toxic activity on human normal fibroblast cell line at a dose of 20 ng. 

According to obtained results, anticancer activity of hadonin is of high pharmaceutical value to follow its therapeutic potency in animal models. 

**Figure 1.**
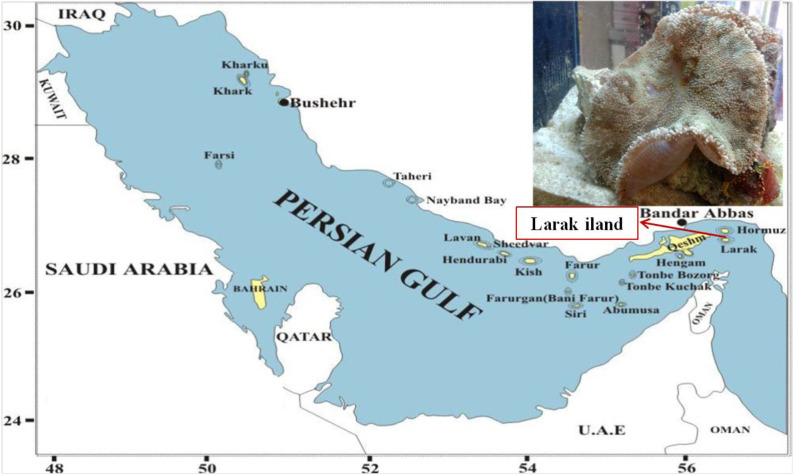
Specimen collection area. Larak Island, the Persian Gulf (26°51'12" N 56°21'20" E).

**Figure 2:**
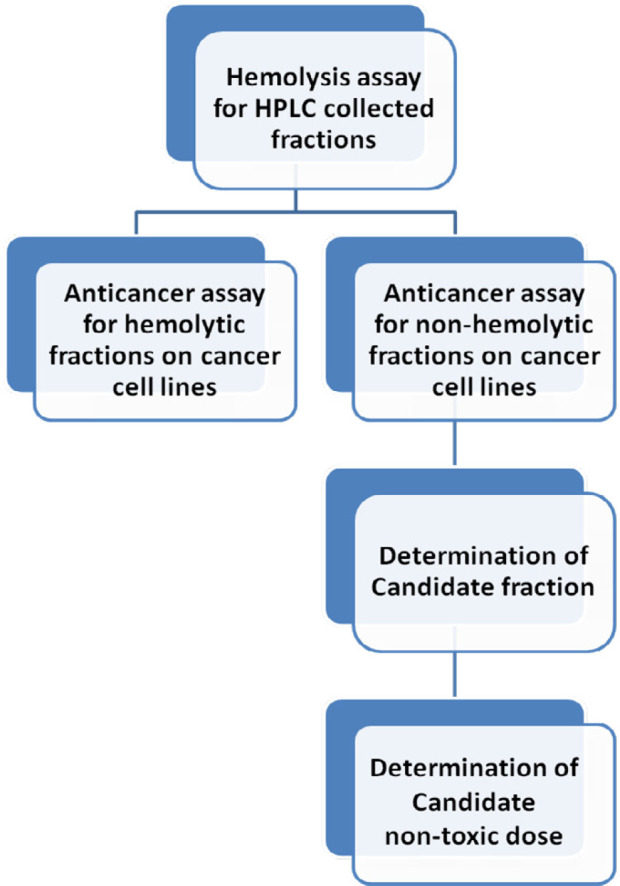
Demonstration of** c**lassified goals to reaching a non-hemolytic non-toxic anticancer fraction

**Figure3:**
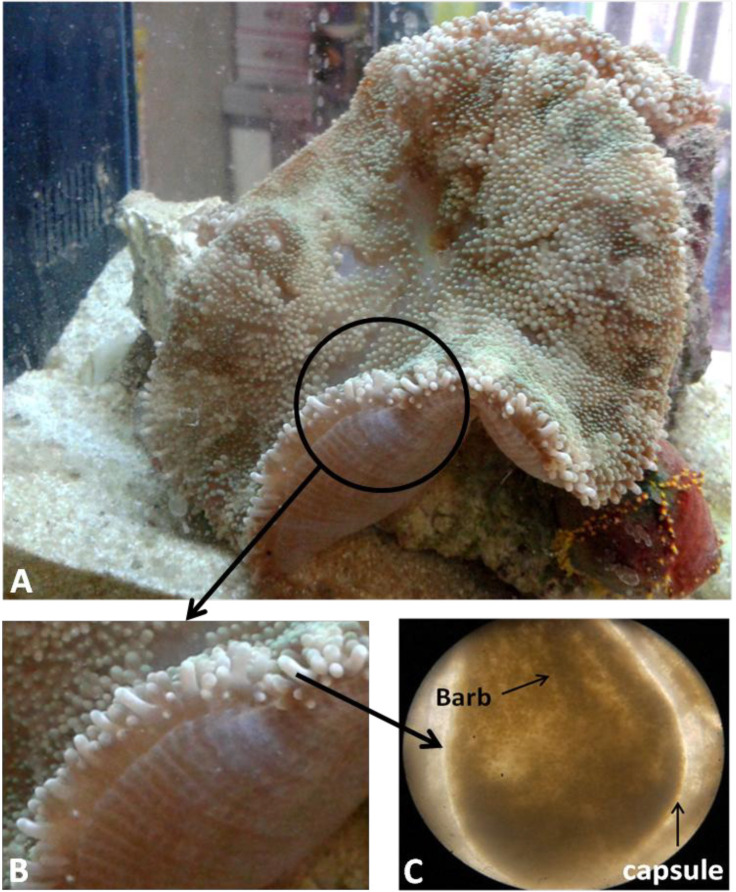
Macroscopic and microscopic image of the tentacles of *Stichodactyla haddoni*. A. Macroscopic image. B. Magnified lateral view of tentacles. C. Microscopic image of a tentacle containing capsule and barb (C: 40X).

**Figure 4:**
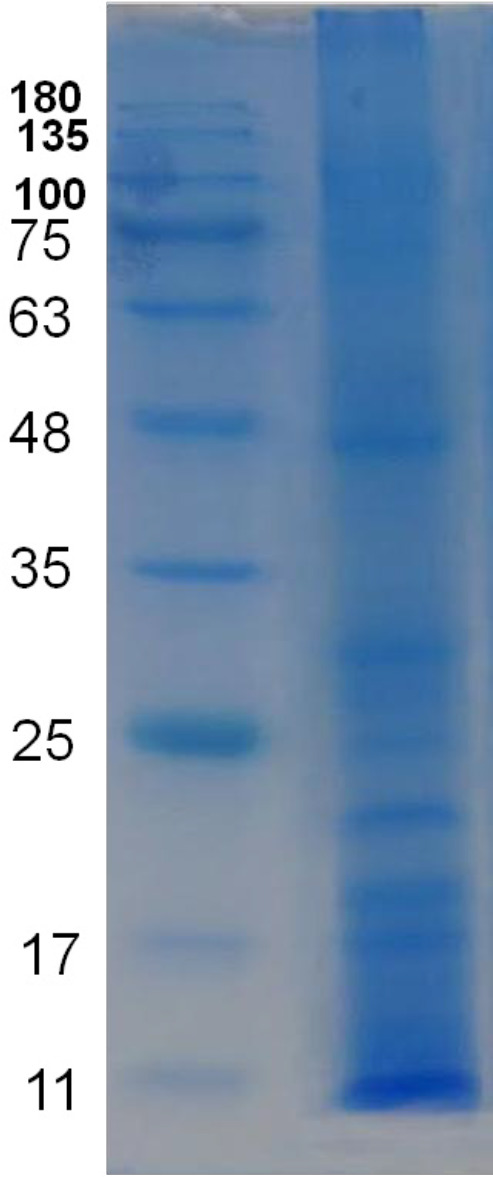
Protein profile of water extract of the venom of *S. haddoni*. SDS-PAGE results showed about 12 separate bands and molecular weight of observed proteins ranged approximately from 8 to 250 kDa

**Figure 5:**
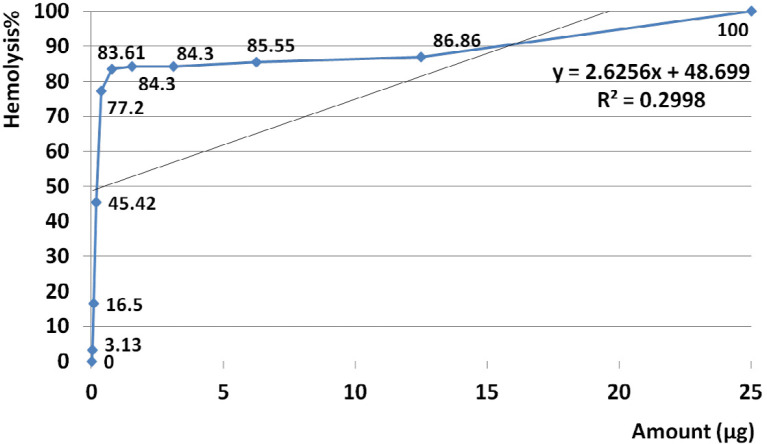
Hemolytic activity of water extracts *S. haddoni* crude venom. The amount of 25 µg crude venom produced 100% hemolysis and HD50 identified at 0.5 µg. Based on regression analysis, no general linearity was seen at examined amounts of venom ranged from 0.023 to 25 µg. This indicates that hemolysis activity was generally not dose dependent at estimated range (R2= 0.299).

**Figure 6:**
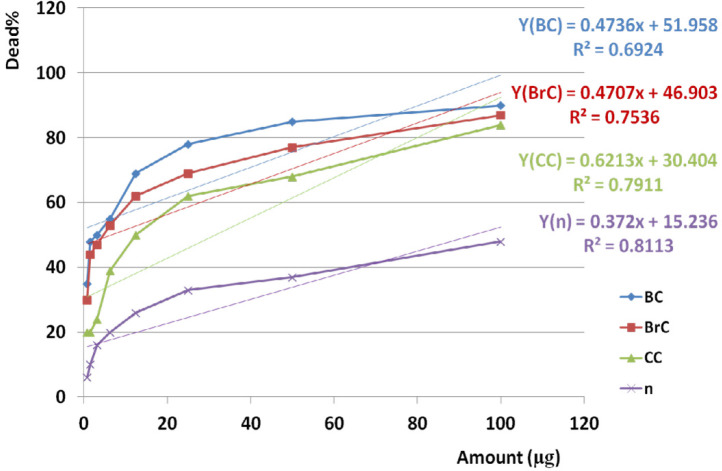
Anticancer activities of the crude extract against cancer and normal cell lines during 24 h. EC50 of crude venom against cancer and normal cell lines was observed at 4.13, 6.58, 31.54, and 93.45 µg respectively. Linear regression analysis showed significant correlation between anticancer activity of crude venom on BC, BrC, and CC and n cell lines and examined doses (R2BC = 0.692, R2BrC = 0.754, R2CC = 0.791, R2n = 0.811). “BC”, “Brc”, “CC”, and “n” are abbreviations of Breast Cancer, Brain Cancer, Colon Cancer, and Normal cell line

**Figure 7:**
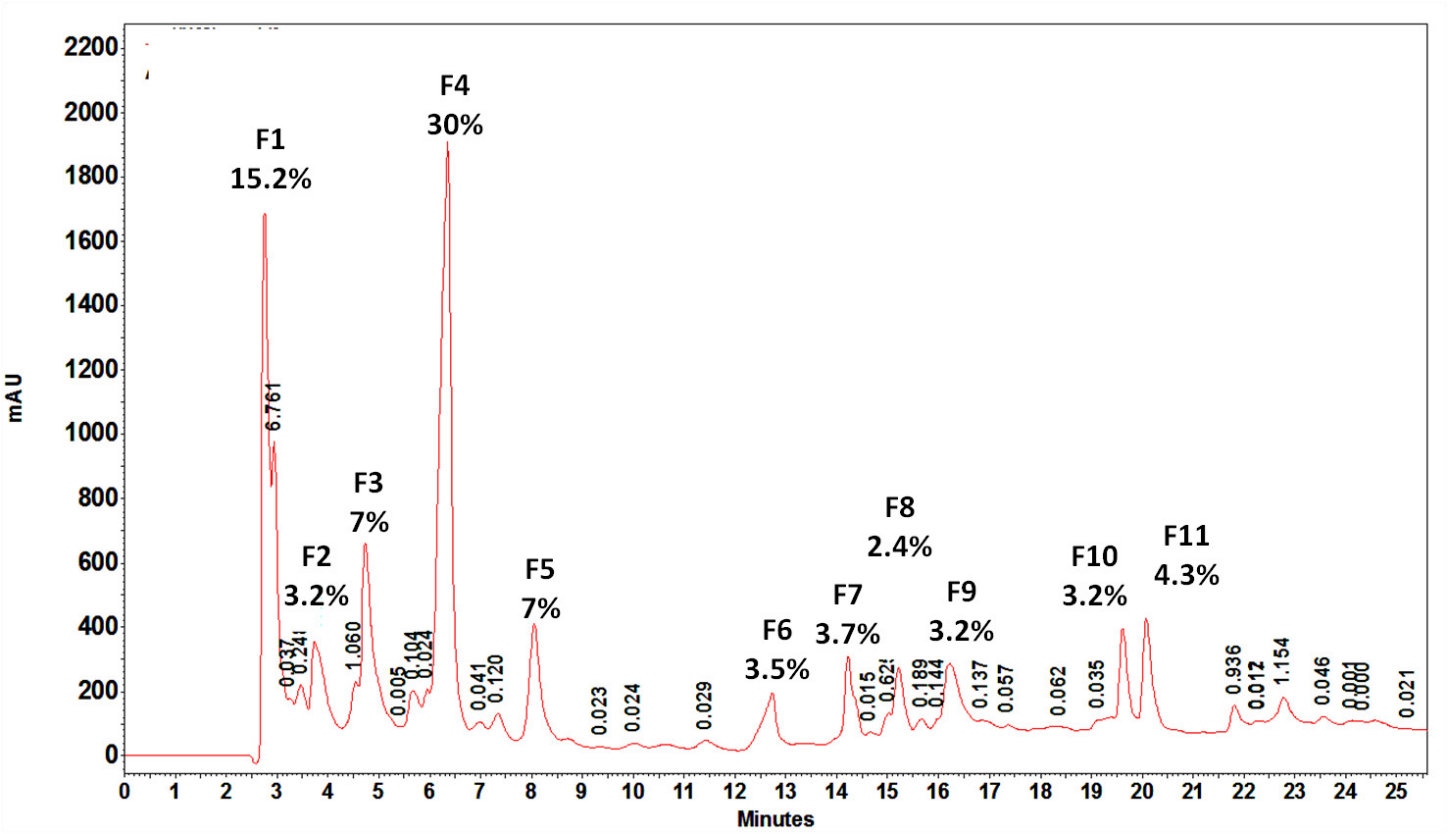
HPLC profile of water extract of tentacles removed from *S. haddoni*. 11 major and minor peaks were seen and area percent greater than 3% collected to further investigations. F4 had the greatest are percent

**Figure 8:**
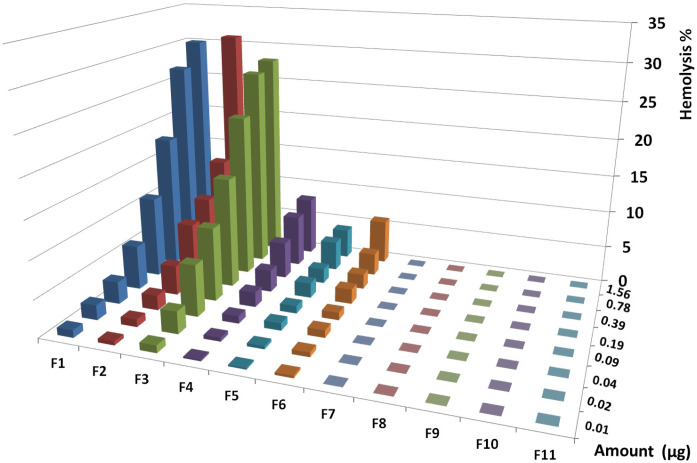
*houres*hemolytic activity of collected HPLC fractions on human RBCs

The highest activities of the first six fractions on human RBCs were equal to 30, 31, 28, 8, 4, and 6% at 1.56 µg respectively. The fractions from F7 to F11 showed no hemolytic activity at all amounts. Regression analysis was showed significant correlation between hemolysis activity of each fraction and examined doses (R2F1 = 0.84, R2F2 = 0.97, R2F3 = 0.71, R2F4 = 0.83, R2F5 = 0.79, R2F6 = 0.96).

**Figure 9:**
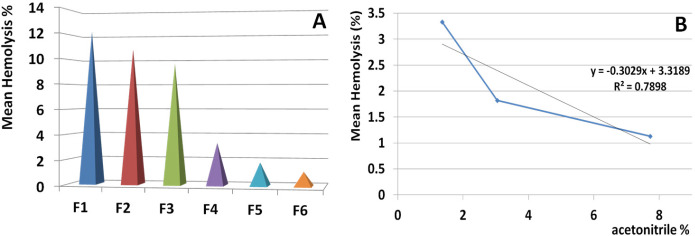
Correlation of hemolytic activity and hydrophobicity of isolated fractions. Linear regression test was performed to control the correlation between activity of hemolytic fraction and the percent of acetonitrile in which the fraction eluted. The result indicated a meaningful correlation (R2 = 0.789). This issue indicated that hydrophobic proteins have the lowest toxicity for human RBCs and also the hydrophobicity is in accordance with no hemolytic activity

**Figure 10:**
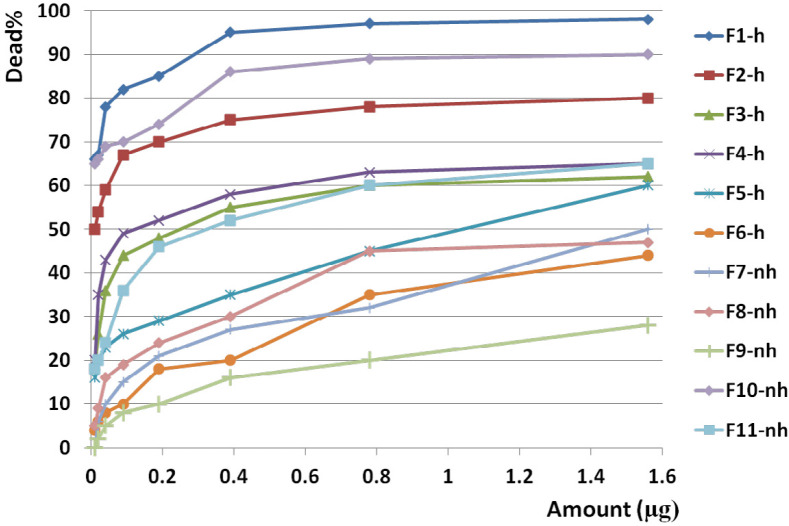
Anticancer activity of all fractions from F1 to F11 on breast cancer cell line. Anticancer activity of examined doses of all fractions against breast cancer cells were correlated significantly together by linear regression analysis (R2= 0.529 - 0.95). Cross comparison of the mean of anticancer activity of each fraction with the other fractions by Paired sample *t*-test showed significant differences in almost all isolated fractions against BC cells (*p* value < 0.05).

**Figure 11:**
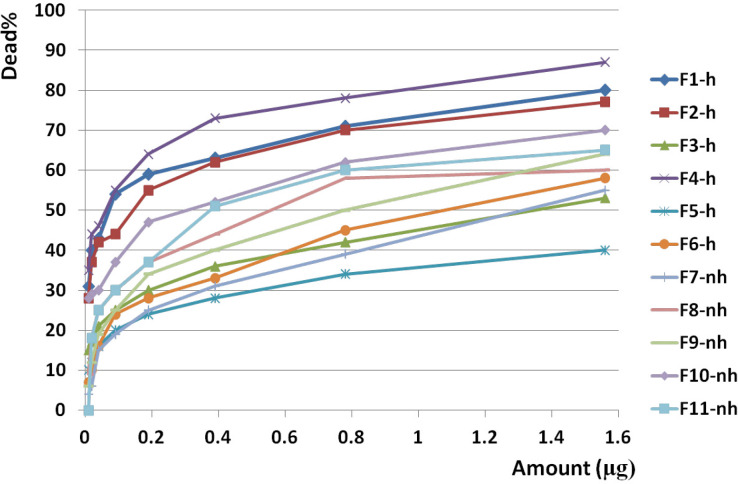
Anticancer activity of all fractions from F1 to F11 on brain cancer cell line. Anticancer activity of examined doses of all fractions against brain cancer cells were correlated significantly together by linear regression analysis (R2= 0.679 - 0.868). Cross comparison of the mean of anticancer activity of each fraction with the other fractions by Paired sample t test showed significant differences in almost all isolated fractions against BrC cells (*p* value < 0.05).

**Figure 12:**
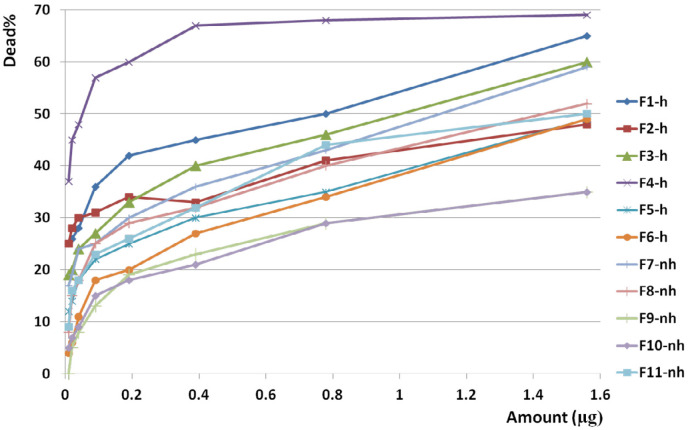
Anticancer activity of all fractions from F1 to F11 on colon cancer cell line. Anticancer activity of examined doses of all fractions against colon cancer cells were correlated significantly together by linear regression analysis (R2= 0.528 - 0.945). Cross comparison of the mean of anticancer activity of each fraction with the other fractions by Paired sample *t*-test showed significant differences in almost all isolated fractions against CC cells (*p* value < 0.05)

**Figure 13:**
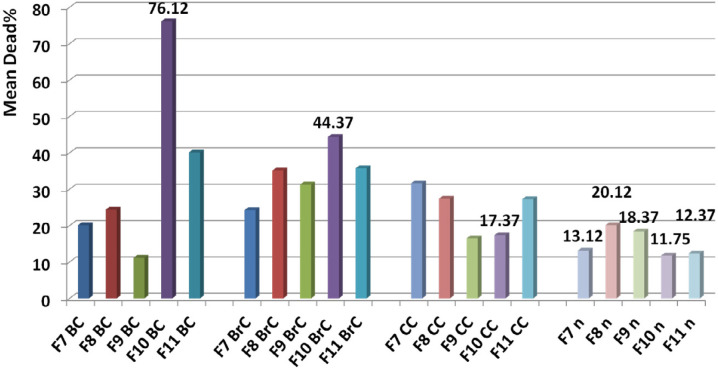
Determination of candidate fraction. Comparison of toxicity of F9, F10, and F11 on normal cell with the other cancer cells showed a significant difference based on paired sample student *t*-test (*P* value < 0.05) while F7 and F8 had similar toxicity with F10 and F11, and F9 respectively (*P* value > 0.05). Comparing the mean toxicity of the fractions between together showed that F10 had the least toxicity and selected as candidate fraction

**Figure 14:**
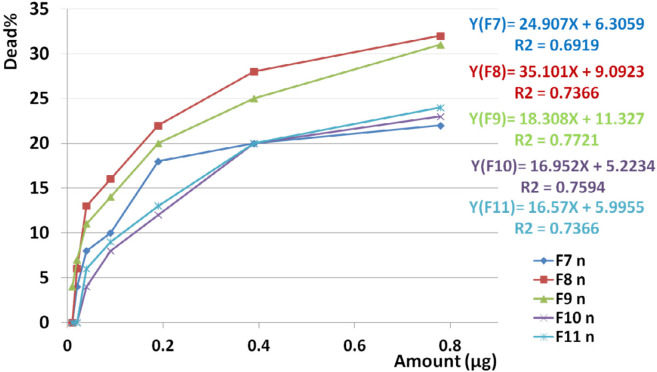
Toxicity of non-hemolytic fractions on normal cell line. Linear regression analysis showed significant correlation between toxicity of F7, F8, F9, F10, and F11 on normal cell lines with increasing doses of the fraction (R2F7 = 0.7, R2F8 = 0.73, R2F9 = 0.772, R2F10 = 0.759, R2F11 = 0.736).

**Figure 15:**
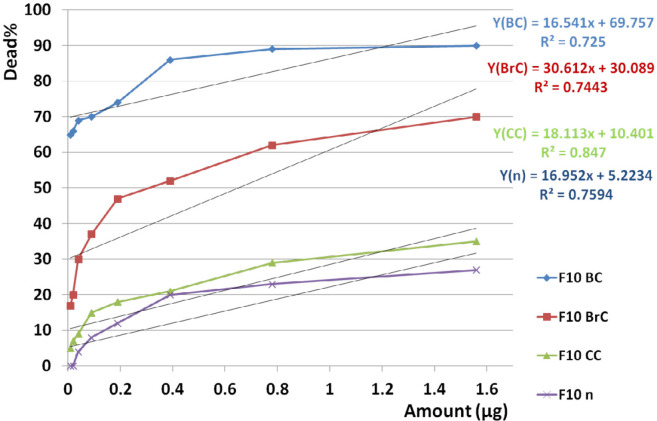
Dose dependency of the candidate fraction, F10, on cancer and normal cell lines. According to linear regression analyses, toxicity of F10 on all three cancer cell lines was dose dependent (R2BC = 0.725, R2BrC = 0.744, R2CC = 0.847) as well as on normal cell line (R2n = 0.759). It means that with decreasing the doses, there is a good chance to access a non-toxic dose on normal cell since the mean toxicity of F10 on cancerous cell is remarkably upper than normal cells

**Figure 16:**
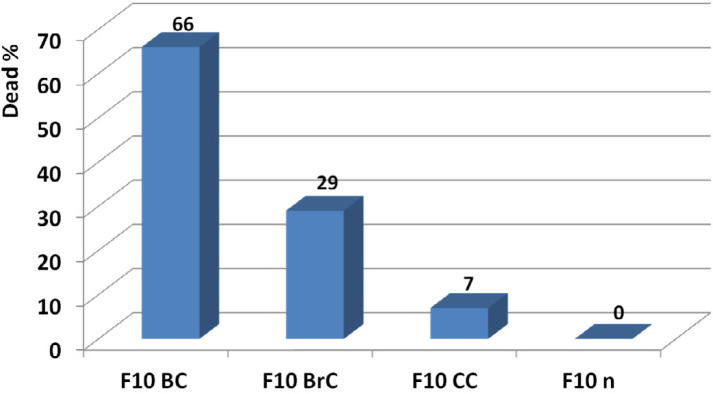
Determination of effective candidate non-toxic dose of F10 and comparison of its activity on breast, brain, and colon cancer cell lines. According to results, F10 showed the best anticancer activity on breast cancer cell line at 20 ng. At this amount no toxicity was observed on normal fibroblast human cell line

**Figure 17:**
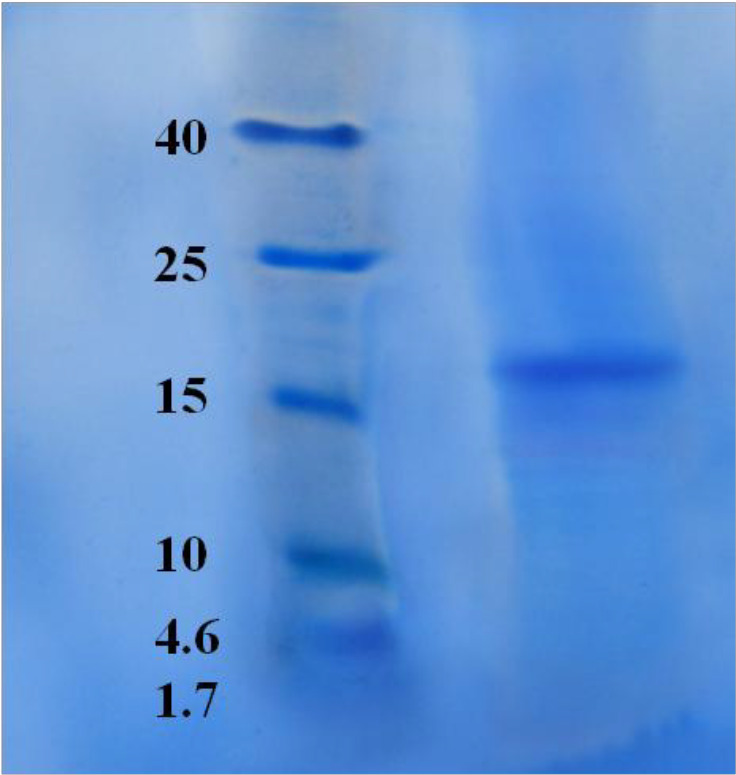
SDS-PAGE for HPLC purified candidate fraction, F10. SDS-PAGE showed one pure band corresponding to molecular weight approximately at 17.5 kDa

## Conclusion

In conclusion, the venom of the Persian Gulf sea anemone contains a potential anticancer agent with reasonable activity against three kinds of cancer cell lines with no toxicity at nanogram level on normal cells. 

## References

[1] Kirthi C, Afzal A, Reddy M, Ali SA, Yerramilli C, Sharma S (2014). A study on the adverse effects of anticancer drugs in an oncology center of a tertiary care hospital. Inter. J. Pharmacy. Pharmaceut. Sci..

[2] Üskent N, Demirbas S, Turken O, Yildirim Ş, Tecimer C, Kandemir G, Yaylaci M (2003). Survival from the precocious brain metastasis of the colon cancer. Turk. J. Cancer.

[3] Aslam MS, Naveed S, Ahmed A, Abbas Z, Gull I, Athar MA (2014). Side effects of chemotherapy in cancer patients and evaluation of patients opinion about starvation based differential chemotherapy. J. Cancer Ther..

[4] Malve H (2016). Exploring the ocean for new drug developments: Marine pharmacology. J. Pharm. Bioallied. Sci..

[5] Cheung RCF, Ng TB, Wong JH (2015). Marine Peptides: Bioactivities and Applications. Mar. Drugs.

[6] Michael TDC, Clinton GLV (2015). Recent Advances in Drug Discovery from South African Marine Invertebrates. Mar. Drugs.

[7] Hu Y, Chen J, Hu G, Yu J, Zhu X, Lin Y, Chen S, Yuan J (2015). Statistical Research on the Bioactivity of New Marine Natural Products Discovered during the 28 Years from 1985 to 2012. Mar. Drugs.

[8] Harvey AL (2014). Toxins and drug discovery. Toxicon.

[9] Martins RD, Alves RS, Martins AMC, Barbosa PSF, Evangelista JSAM, Evangelista JJF, Ximenes RM, Toyama MH, Toyama DO, Souza AJF, Orts DJB, Marangoni S, de Menezes DB, Fonteles MC, Monteiro HSA (2009). Purification and characterization of the biological effects of phospholipase A2 from sea anemone Bunodosoma caissarum. Toxicon.

[10] Mariottini GL, Pane L (2014). Cytotoxic and cytolytic cnidarian venoms A review on health implications and possible therapeutic applications. Toxins.

[11] Newman DJ, Cragg MG (2013). Marine natural products and related compounds in clinical and advanced preclinical trials. Anti-Cancer Agents Med. Chem..

[12] Petit K, Biard JF (2013). Marine Natural products and related compounds as anticancer agents: an overview of their clinical status. Anti-Cancer Agents Med. Chem..

[13] Essack M, Bajic VB, Archer JA (2012). Conotoxins that confer therapeutic possibilities. Mar. Drugs.

[14] Chi V, Pennington MW, Norton RS, Tarcha EJ, Londono LM, Sims-Fahey B, Upadhyay SK, Lakey JT, Iadonato S, Wulff H, Beeton C, Chandy KG (2012). Development of a sea anemone toxin as an immunomodulator for therapy of autoimmune diseases. Toxicon.

[15] Molinski TF, Dalisay DS, Lievens SL, Saludes JP (2009). Drug development from marine natural products. Nat .Rev. Drug Discov..

[16] Leal M, Sapra P, Hurvitz SA, Senter P, Wahl A, Schutten M, Shah DK, Haddish-Berhane N, Kabbarah O (2014). Antibody-drug conjugates: An emerging modality for thetreatment of cancer. Ann. NY Acad. Sci..

[17] Rinehart KL, Holt TG, Fregeau NL, Stroh JG, Keifer PA, Sun F, Li LH, Martin DG (1990). Ecteinascidins 729, 743, 745, 759A, 759B, and 770: potent antitumor agents from the Caribbean tunicate Ecteinascidia turbinate. J. Org. Chem..

[18] Martindale (2009). The Complete Drug Reference (database on the internet). Cytarabine. Thomson MICROMEDEX.

[19] Lichtman MA (2013). A historical perspective on the development of the cytarabine (7 days) and daunorubicin (3 days) treatment regimen for acute myelogenous leukemia: 2013 the 40th anniversary of 7 + 3. Blood Cells Mol. Dis..

[20] Shen W, Kim JS, Kish PE, Zhang J, Mitchell S, Gentry BG, Breitenbach JM, Drach JC ans Hilfinger J (2009). Design and synthesis of Vidarabine prodrugs as antiviral agents. Bioorg. Med. Chem. Lett..

[21] Cuadrado A, Garcia-Fernandez LF, Gonzalez L, Gonzalez L, Suarez Y, Losada A, Alcaide V, Martinez T, Fernandez-Sousa JM, Sanchez-Puelles JM, Munoz A (2003). Aplidin induces apoptosis in human cancer cells via glutathione depletion and sustained activation of the epidermal growth factor receptor, Src, JNK, and p38 MAPK. J. Biol. Chem..

[22] Sudek S, Lopanik NB, Waggoner LE, Hildebrand M, Anderson C, Liu H, Patel A, Sherman DH, Haygood MG (2007). Identification of the putative bryostatin polyketide synthase gene cluster from ―Candidatus Endobugula sertula‖, the uncultivated microbial symbiont of the marine bryozoan Bugula neritina. J. Nat. Prod..

[23] Talpir R, BenayahuY, Kashman L, Pannell L, Schleyer M (1994). Hemiasterlin and geodiamolide TA: two new cytotoxic peptides from the marine sponge Hemiasterella minor. Tetra Lett..

[24] LingYH, Aracil M, Jimeno J, Perez-Soler R, Zou1 Y (2009). Molecular pharmacodynamics of PM02734 (elisidepsin) as single agent and in combination with erlotinib; synergistic activity in human non-small cell lung cancer cell lines and xenograft models. Eur. J. Cancer.

[25] Ramezanpour M, Burke da Silva K, Sanderson BJS (2012). Differential susceptibilities of human lung, breast and skin cancer cell lines to killing by five sea anemone venoms. J. Venom. Anim. Toxins: incl Trop. Dis..

[26] Monroy-Estrada H, Chirino Y, Soria-Mercado IE, Sánchez-Rodríguez J (2013). Toxins from the Caribbean Sea anemone Bunodeopsis globulifera increase cisplatin-induced cytotoxicity of lung adenocarcinoma cells. J Venom Anim Toxins: incl Trop. Dis..

[27] Soletti RC, de Faria GP, Vernal J, Terenzi H, Anderluh G, Borges HL, Moura-Neto V, Gabilan NH (2008). Potentiation of anticancer-drug cytotoxicity by sea anemone pore-forming proteins in human glioblastoma cells. Antican. Drugs.

[28] Anderluh G, Maček P (2002). Cytolytic peptide and protein toxins from sea anemones (Anthozoa:Actiniaria). Toxicon.

[29] Maček P, Zecchini M, Stanek K, Menestrina G (1997). Effect of membrane partitioned n-alcohols and fatty acids on pore-forming activity of a sea anemone toxin. Eur. Biophys. J..

[30] Tejuca M, Dalla Serra M, Potrich C, Alvarez C, Menestrina G (2001). Sizing the radius of the pore formed in erythrocytes and lipid vesicles by the toxin Sticholysin I from the sea anemone Stichodactyla helianthus. J. Membr. Biol..

[31] Tejuca M, Anderluh G, Dalla Serra M (2009). Sea anemone cytolysins as toxic components fo immnunotoxins. Toxicon.

[32] Tejuca M, Pérez-Barzaga V, Pazos F, Álvarez C, Lanio ME (2009). Construction of sea anemone cytolysin-based immunotoxins for selective killing of cancer cells. Rev. Cub. Fisica..

[33] Fedorov S, Dyshlovoy S, Monastyrnaya M, Shubina L, Leychenko E, Kozlovskaya E, Jin JO, Kwak JY, Bode AM, Dong Z, Stonika V (2010). The anticancer effects of actinoporin RTX-A from the sea anemone Heteractis crispa (=Radianthus macrodactylus). Toxicon.

[34] Jiang X, Chen H, Yang W, Liu Y, Liu W, Wei J, Tu H, Xie X, Wang L, Xu A (2003). Functional expression and characterization of an acidic actinoporin from sea anemone Sagartia rosea. Biochem. Biophys. Res. Commun..

[35] Yan L, Herrington J, Goldberg E, Dulski PM, Bugianesi RM, Slaughter RS, Banerjee P, Brochu RM, Priest BT, Kaczorowski GJ, Rudy B, Garcia ML (2005). Stichodactyla helianthus peptide, a pharmacological tool for studying Kv3 2 channels. Mol. Pharmacol..

[36] Frazão B, Vasconcelos V, Antunes A (2012). Sea Anemone (Cnidaria, Anthozoa, Actiniaria) Toxins. An Overview. Mar. Drugs.

[37] Kem WR, Pennington MW, Norton RS (1999). Sea anemone toxins as templates for the design of immunosuppressant drugs. Perspect. Drug Discov. Des..

[38] Laemmli UK (1970). Cleavage of structural proteins during the assembly of the head of bacteriophage T4. Nature.

[39] Memar B, Jamili S, Shahbazzadeh D, Pooshang Bagheri K (2016). The first report on coagulation and phospholipase A2 activities of Persian Gulf lionfish, Pterois russelli, an Iranian venomous fish. Toxicon.

[40] Mosmann T (1983). Rapid colorimetric assay for cellular growth and survival: application to proliferation and cytotoxicity assays. J. Immunol. Methods.

[41] Subramanian B, Sangappellai T, Rajak CR, Diraviam B (2011). Pharmacological and biomedical properties of sea anemones Paracondactylis indicus, Paracondactylis sinensis, Heteractis magnifica and Stichodactyla haddoni from East coast of India. Asian Pacific J. Trop. Med..

[42] Sudharsan S, Seedevi P, Kanagarajan U, Dalvi SR, Guptha S, Poojary N, Shanmugam V, Srinivasan A, Shanmugam A (2013). Analgesic and neuromodulatory effects of sea anemone Stichodactyla mertensii (Brandt, 1835) methanolic extract from southeast coast of India. Afric J. Pharm. Pharmacol..

[43] Marino A, Morabito R, La Spada G (2009). Factors altering the haemolytic power of crude venom from Aiptasia mutabilis (Anthozoa) nematocysts. Comp BiochemPhysiol. A..

[44] Cline EI, Wiebe LI, Young JD, Samuel J (1995). Toxic effects of the novel protein UpI from the sea anemone Urticina piscivora. Pharmacol. Res..

[45] Ravindran SV, Kannan L, Venkateshvaran K (2010). Biological activity of sea anemons proteins:I Toxicity and histopatology. Indian J. Exp. Biol..

[46] Avila AD, Mateo de Acosta C, Lage A (1988). A new immunotoxin built by linking a hemolytic toxin to a monoclonal antibody specific for immature T lymphocytes. Int. J. Cancer.

[47] Batista U, Macek P, Sedmak B (1990). The cytotoxic and cytolytic activity of Equinatoxin II from the sea anemone Actinia equina. Cell Biol. Int. Rep..

